# Puerarin Exerts Protective Effects on Wear Particle-Induced Inflammatory Osteolysis

**DOI:** 10.3389/fphar.2019.01113

**Published:** 2019-10-01

**Authors:** Chao Yang, Juehong Li, Kechao Zhu, Xiangwei Yuan, Tao Cheng, Yebin Qian, Xianlong Zhang

**Affiliations:** Department of Orthopedics, Shanghai Jiao Tong University Affiliated Sixth People’s Hospital, Shanghai, China

**Keywords:** titanium particles, osteolysis, puerarin, osteoclastogenesis, ERK pathway

## Abstract

Wear particle-stimulated inflammatory bone destruction and the consequent aseptic loosening remain major postoperative problems for artificial joints. Studies have indicated that puerarin promotes osteogenesis and alleviates lipopolysaccharide-induced osteoclastogenesis *in vitro*. However, the underlying molecular mechanism by which puerarin interacts with receptor activator of nuclear factor kappa-B ligand (RANKL)-mediated osteoclast formation *in vitro* and wear particle-stimulated osteolysis *in vivo* has not been reported. In this work, the protective effects exerted by puerarin on titanium particle-stimulated bone destruction *in vivo* and on RANKL-induced osteoclast activation in osteoclastic precursor cells *in vitro* were investigated. As expected, puerarin significantly inhibited wear particle-mediated bone resorption and proinflammatory cytokine productions in a calvarial resorption model. Additionally, puerarin inhibited RANKL-induced osteoclast activation, bone resorption ability, and F-actin ring formation *in vitro* as puerarin concentration increased. Furthermore, mechanistic investigation indicated that reduced RANKL-stimulated MEK/ERK/NFATc1 signaling cascades might regulate the protective effect of puerarin. Conclusively, these results indicate that puerarin, a type of polyphenol, might serve as a protective agent to prevent osteoclast-related osteolytic diseases.

## Introduction

Artificial joint replacement has achieved great success in orthopedics and is considered the most effective surgical treatment for various end-stage hip and knee diseases (Goodman et al., 2014). However, wear debris-stimulated bone destruction and the subsequent implant loosening remain major postoperative complications for artificial joints (Bozic et al., 2009; Prokopetz et al., 2012). Wear debris, generated from the surface of implant materials, are responsible for the activation of the immune system and inflammatory reactions, which subsequently promote the secretion of proinflammatory cytokines (Rao et al., 2012; Gallo et al., 2013; Yang et al., 2016). These inflammatory cytokines can enhance osteoclast recruitment and function, thus interrupting the balance between osteogenesis and osteoclastogenesis (Murata et al., 2017; Ping et al., 2017b). Therefore, an agent that inhibits inflammatory cytokines releases and the activation of osteoclastic function is a candidate for the protection and prevention of pathological osteolytic diseases.

Although the pathological osteolytic process involves a variety of cells, osteoclasts are multinucleated giant bone-destructing cells derived from the hematopoietic monocyte/macrophage linage. Furthermore, osteoclasts induces osteolysis, ultimately leading to aseptic loosening (Greenfield et al., 2002; Wu et al., 2018a). Given the important influence osteoclast differentiation, two osteoclast-stimulated cytokines, macrophage colony-stimulating factor (M-CSF) and receptor activator of nuclear factor kappa B ligand (RANKL), are applied to promote osteoclast maturity (Asagiri and Takayanagi, 2007). M-CSF is mainly responsible for the proliferation of osteoclastic precursor cells through binging to its receptor, whereas RANKL is a crucial positive controller of osteoclast function (Mediero et al., 2016). After binding to RANK, RANKL initiates the recruitment of tumor necrosis factor receptor-associated factors (TRAFs), which in turn induce the activation of osteoclastic signaling pathways, such as the mitogen-activated protein kinase (MAPK), nuclear factor kappa B (NF-κB), and phosphatidylinositol 3-kinase/AKT (PI3k/Akt) signaling pathways (Mandal et al., 2009; Guo et al., 2017; Wu et al., 2019). These events are associated with the activation of osteoclastic differentiation and ultimately lead to the upregulation of c-fos and nuclear factor of activated T cells cytoplasmic 1 (NFATc1) expression to induce osteoclastogenesis (Asagiri et al., 2005; Takayanagi, 2007). Therefore, blocking the osteoclast-associated signaling pathways may be an alternative approach for treating wear particle-stimulated bone destruction.

Due to their pleiotropic action and fewer side effects, natural plant extracts have attracted great attention in the field of wear particle-induced osteolytic diseases. Puerarin, a type of polyphenol and the main isoflavone glucoside isolated from the plant Pueraria lobata, exhibits beneficial effects due to its antioxidant, anti-inflammatory, anti-diabetes and anti-apoptotic properties (Xiao et al., 2011; Zhang et al., 2011; Yuan et al., 2014; Deng et al., 2017). Recent studies suggested that puerarin confers a protect effect by inhibiting osteoclastogenesis in osteoporotic rats and promotes new bone formation in osteoblast implants (Liu et al., 2016; Yang et al., 2018). Moreover, puerarin inhibits inflammation and regulates proinflammatory cytokines secretion, which is associated with the occurrence of inflammatory osteolysis (Xiao et al., 2011). In addition, there is evidence that puerarin also inhibits osteoclast differentiation from osteoclast precursor cells and attenuates lipopolysaccharide-stimulated bone destruction *in vivo* (Zhang et al., 2016; Park et al., 2017). However, the underlying mechanism by which puerarin mitigates RANKL-mediated osteoclast differentiation and function at the cellular level and alleviates wear debris-stimulated inflammatory bone destruction in a calvarial resorption model has not been investigated.

Thus, the purpose of this work was to evaluate the protective effects of puerarin against titanium debris-stimulated inflammatory bone destruction *in vivo* and *in vitro*, as well as to examine its effect and underlying mechanism during osteoclast formation. We first established a calvarial osteolysis model *in vivo*. Micro-computed tomography (micro-CT) and histological analysis were applied to evaluate the protective effect exhibited by puerarin. Then, we assessed the direct inhibitory effect of puerarin on RANKL-stimulated formation and resorption pits of osteoclastic precursor cells *in vitro*, and the potential mechanisms were further explored by verifying the affected signaling pathways as described above.

## Materials and Methods

### Puerarin and Particle Preparation

Puerarin (purity ≥98.0%, MW: 416.38) was purchased from Sigma-Aldrich (St. Louis, USA). Ti particles were obtained from Johnson Matthey Chemical (MA, USA). The particles were washed in 70% ethanol and sterilized at 180 °C for 6 h. Ti particles were endotoxin-negative, as verified by a Limulus amebocyte lysate assay (Biowhittaker, USA) (Frellsen et al., 2016).

### Mouse Calvarial Osteolysis Model

The animal experiment was approved by the Animal Care Committee of Shanghai Jiao Tong University Affiliated Sixth People’s Hospital and strictly followed the principles of the Laboratory Animals Care and Use. First, 32 pathogen-free and healthy male C57BL/6J mice (6–8 weeks old) were purchased from the Experimental Animal Center of Shanghai Sixth People’s Hospital. Animals were assigned randomly into the following groups (n = 8/group): the sham group and vehicle groups were subjected to phosphate buffered saline (PBS) and Ti particles, respectively, whereas mice in the experimental groups were subjected to Ti particles and different dosages of puerarin (low and high dosage groups).

As previously described (Wang et al., 2015; Li et al., 2018), we used a calvarial resorption model to investigate the inhibitory abilities of puerarin on Ti particle-induced bone resorption and bone destruction *in vivo*. Four percent chloral hydrate was applied to anesthetize the mice by intraperitoneal injection. A 1 cm length incision was made in the middle of the mouse head. Then, the cranial periosteum was cut and separated from the calvaria, and 20 mg Ti particles were placed on the calvarial surface in the positive control and experimental groups, whereas mice in the negative control group were subjected to operation without Ti particle treatment. In the low-dose and high-dose groups, puerarin was intraperitoneally injected daily at 10 mg/kg/day and 50 mg/kg/day, respectively, for 14 days. Mice in the sham control or positive control groups were injected with sterile PBS daily. Fourteen days after surgery, the animals were sacrificed with an overdose of anesthesia. Mouse skulls were collected for further assessment.

### Micro-CT Scanning

After harvesting and fixation, micro-CT (SkyScan 1172, Bruker micro CT, Karlsdorf-Neuthard, Germany) was conducted at a resolution of 9 mm to scan mouse calvariae, and the associated analysis software was applied for data analysis. Representative images of the calvariae were reconstructed and analyzed using the NRecon software (SkyScan, Port Richey, FL, USA). A cylindrical region of interest (3×3×1 mm) was selected as previously described (Li et al., 2017). CT Analyzer Software (Version: 1.15.4.0+, Bruker) was applied to measure and analyze the bone mineral density (BMD), bone volume against tissue volume (BV/TV), total porosity, and the number of pores.

### Histological and Immunohistochemical Analysis

After fixing in formalin for 2 days, 10% ethylenediaminetetraacetic acid (EDTA) was applied to decalcify mouse calvariae for 21 days, and paraffin wax was used to embed the samples. Sections OF 5 μm were cut in the coronal plane on a microtome, deparaffinized, and rehydrated. Hematoxylin and eosin (H&E), Masson’s trichrome, and tartrate-resistant acid phosphatase (TRAP) staining (Sigma) were performed. The stained sections were photographed by a light microscope (Leica, Wetzlar, Germany). The area of erosion (mm^2^), number of osteoclasts, and percentage of osteoclasts per bone surface (OCs/BS, %) of each group were assessed and measured by Image Pro Plus software 6.0 (Media Cybernetics, Bethesda, MD, USA).

For immunochemical staining of tumor necrosis factor alpha (TNF-α), interleukin (IL)-1β, and extracellular signal-regulated kinase1/2 (ERK1/2) the remaining sections were fixed and subjected to a water bath for antigen retrieval. Sections were blocked and then incubated with antibodies for TNF-α, IL-1β or ERK1/2 overnight at 4 °C. Then, the sections were rinsed and incubated with secondary antibodies for 60 min and stained with hematoxylin.

### Cell Culture and Induction

To separate and culture the osteoclastic precursor cells, bone marrow-derived macrophages (BMMs) were obtained from the femurs of 6-week-old healthy male C57BL/6J mice (Li et al., 2018). Briefly, complete α-minimum essential medium (α-MEM, HyClone) containing 10 ng/mL M-CSF (PeproTech, NJ, USA) was used to culture the BMMs. The next day, cell suspensions were obtained, centrifuged, and resuspended in complete medium with 30 ng/mL M-CSF. After culturing for 72 h, the osteoclastic precursor cells were scraped and collected for further study.

RAW264.7 cells were obtained from the Type Culture Collection of the Chinese Academy of Sciences (Shanghai, China) and cultured in complete Dulbecco’s modified Eagle medium (DMEM, HyClone). The cells were refreshed every 2 days and passaged at approximately 80% confluence by scraping.

### Cell Proliferation

The proliferation of puerarin-treated BMMs was assessed by Cell Counting Kit-8 (CCK-8) assay. The cells (2 × 10^4^ cells/well) were added to a 96-well plate and incubated with complete medium containing 30 ng/ml M-CSF for 1 day. The next day, BMMs were cultured in α-MEM medium with various puerarin concentrations (0, 1, 2, 5, 10, 20, 25, 50, 100, or 200 μM), M-CSF (30 ng/ml) for 72 h. The cells were washed 3 times and cultured for another 3 h with 10% CCK-8-containing medium. A microplate reader was used to evaluate cell viability at 450 nm.

### Osteoclast Differentiation of Osteoclastic Precursor Cells *in Vitro*

BMMs were used to assess osteoclast formation ability. Briefly, the cells were cultured and induced in α-MEM medium with the addition of M-CSF (30 ng/ml), RANKL (100 ng/ml) and 0, 1, 5, or 25 μM puerarin for 7 days. In addition, BMMs were incubated in α-MEM medium containing M-CSF (30 ng/ml) and RANKL (100 ng/ml) with 25 μM puerarin for days 0–2, 1–3, 2–4, 3–5. On day 7, the cells were fixed and washed 3 times, then subjected to TRAP (Sigma) staining to investigate osteoclast differentiation of osteoclastic precursor cells. TRAP-positive cells (≥3 nuclei) were considered typical osteoclasts, and observed by a light microscope (Leica).

To investigate whether ERK1/2 pathway is indeed responsible for puerarin treated efficacy, ERK1/2 agonist Honokiol (20 μM) (MedChem Express, USA) was used to combination with puerarin. The cells were induced and fixed at day 7, then subjected to TRAP staining to evaluate the inhibitory effect of puerarin on ERK pathway.

### F-Actin Ring Formation by Fluorescence Assay

Fluorescence staining was preformed to confirm the inhibitory effect of puerarin on mature F-actin ring formation. The cells were induced and cultured in α-MEM medium with the addition of M-CSF (30 ng/ml), RANKL (100 ng/ml) and 0, 1, 5, or 25 μM puerarin. When the mature osteoclasts were observed on day 7, BMMs were fixed and permeabilized with 0.1% Triton X for 15 min, respectively, and then stained with phalloidin diluted in 1% bovine serum albumin for 30 min. Then, the cells were rinsed 3 times, subjected to DAPI for 5 min and observed using a fluorescence microscope (Leica).

### Bone Resorption Assay

An Osteo Assay Plate (OAP; Corning, New York, USA) was applied to evaluate the potential role of puerarin on osteoclastic bone resorption pits. The cells were seeded on the OAP in triplicate at a density of 3 × 10^4^ and cultured in complete medium supplemented with M-CSF and RANKL as described above. When typical osteoclasts were observed on day 4, the complete medium with added M-CSF, RANKL, and 0, 1, 5, or 25 μM puerarin was replaced and cultured for an additional 3 days. On day 7, cells were removed *via* sonication and observed by a light microscope (Leica). The percentage of bone resorption pits was measured by Image Pro Plus.

### Osteoclastic Marker Gene Expression

The expression of osteoclast-related genes was quantified by reverse-transcription polymerase chain reaction (RT-PCR). The cells were induced in complete medium containing M-CSF, RANKL, and various puerarin concentrations (0, 1, 5, or 25 μM) for 5 days. In addition, BMMs were cultured in osteoclast induction medium with or without 25 μM puerarin, and the mRNA expression of osteoclast-related genes on days 1, 3, and 5 was also quantified by RT-PCR. TRIzol reagent (Invitrogen, USA) was applied to extract total RNA. A RevertAid First Strand cDNA Synthesis Kit (Thermo Fisher) was used to synthesize complementary DNA. Quantitative gene analysis was conducted using a FastStart Universal SYBR Green Master (Rox; Roche, Basel, Switzerland) and a PCR instrument (ABI). Gene primers are shown in **Table 1** with GAPDH as a housekeeping gene.

**Table 1:** Primers sequences used for RT-PCR in this study.

Gene	Primer sequences (F: forward; R: reverse; 5′−3′)
Cath-K	F: CTTCCAATACGTGCAGCAGA
	R: TCTTCAGGGCTTTCTCGTTC
CTR	F: ACCGACGAGCAACGCCTACGC
	R: GCCTTCACAGCCTTCAGGTAC
c-fos	F: CCAGTCAAGAGCATCAGCAA
	R: AAGTAGTGCAGCCCGGAGTA
NFATc1	F: CCGTTGCTTCCAGAAAATAACA
	R: TGTGGGATGTGAACTCGGAA
GAPDH	F: ACCCAGAAGACTGTGGATGG
	R: CACATTGGGGGTAGGAACAC

### Western Blotting

RAW264.7 cells were plated and cultured at approximately 80% confluence. Then, the cells were incubated with complete medium containing puerarin (25 μM) for 4 h and subsequently treated with RANKL (100 ng/ml) for a specific period. Afterwards, the cells were scraped, lysed in RIPA lysis duffer containing protease and phosphatase inhibitors (Yeasen, China) for 30 min on ice and subsequently centrifuged. Then, 10% sodium dodecyl sulfate-polyacrylamide gel electrophoresis was applied to separate the total protein, which was subsequently transferred to polyvinylidene fluoride membranes. Membranes were blocked and incubated with antibodies overnight at 4 °C, then, rinsed 3 times and incubated with secondary antibodies for 1 h. Finally, the protein bands were developed using an enhanced chemiluminescence reagent (Millipore, USA). Antibodies of the following were used: ERK1/2, p-ERK1/2, p38 mitogen-activated protein kinase (p38), p-P38, c-jun N-terminal kinase (JNK), p-JNK, phosphatidylinositol 3-kinase/AKT (Akt), p-Akt, NF-κB, p-NF-κB, c-fos, NFATc1, MAP kinase kinase 1/2 (MEK1/2), p-MEK1/2, and β-actin (Cell Signaling Technologies, USA).

### Statistical Analysis

All data are presented as the mean ± standard deviation and are representative of at least three independent experiments. Statistical significance among the groups was analyzed with one-way ANOVA and Student’s t-test using SPSS 17.0. P < 0.05 or P < 0.01 demonstrated significant differences.

## Results

### Puerarin Prevents Wear Particle-Induced Bone Destruction and Resorption *in Vivo*

**Figure 1:**
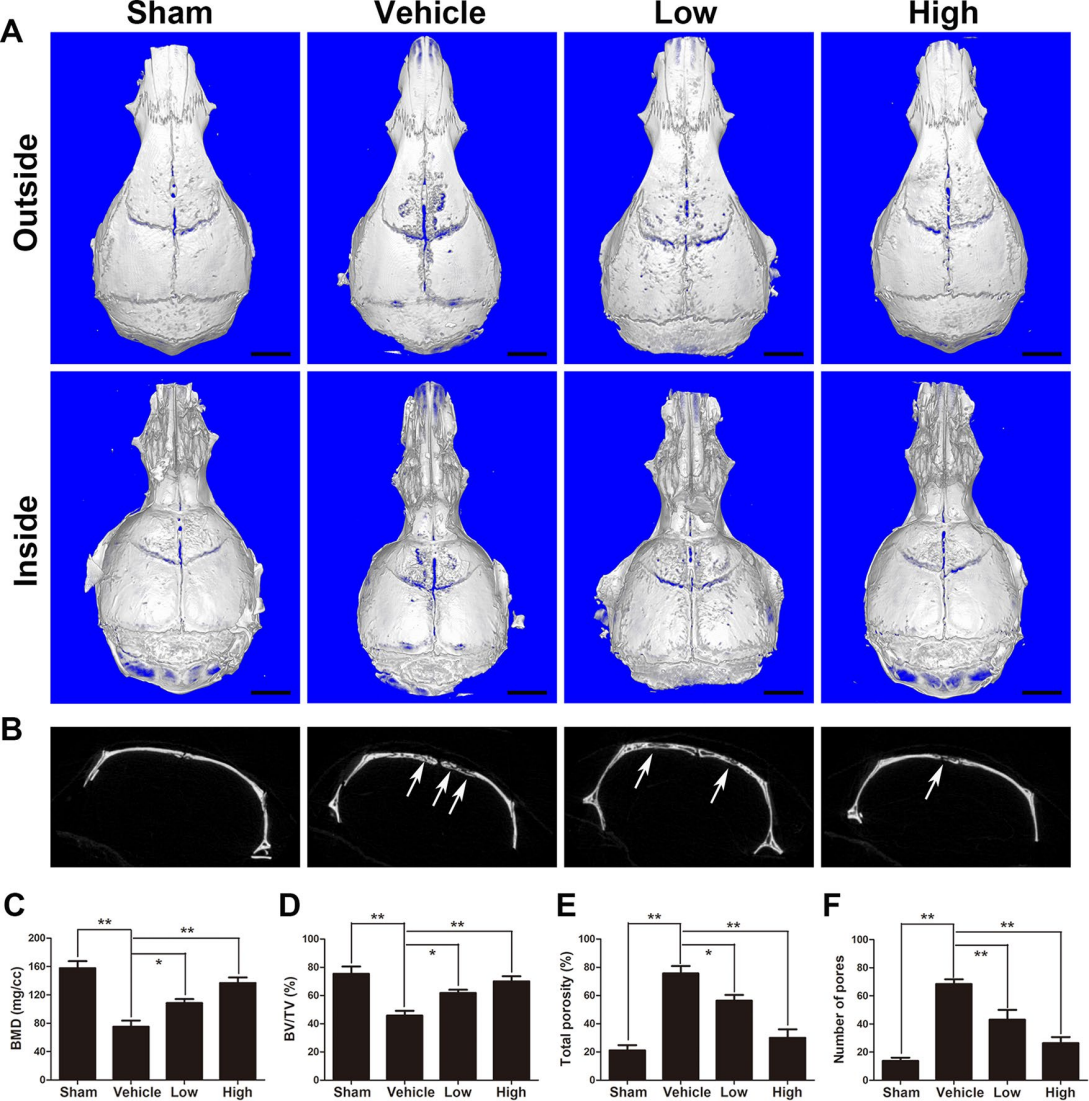
Puerarin attenuates Ti particle-induced osteolysis *in vivo*. **(A)** Representative micro-CT 3D reconstructed images of the calvaria in each group. **(B)** Representative coronal 2D images of the calvaria in each group. The white arrows indicate bone loss. **(C)** bone mineral density (BMD), **(D)** bone volume/total volume (BV/TV), **(E)** total porosity and **(F)** number of pores of each group were measured using micro-CT analyzer software (Bruker). Data are presented as mean ± SD (n= 4/group); *P < 0.05 and **P < 0.01 compared with the vehicle group; Data are representative of at least three independent experiments. Scale bar = 2 mm.

Representative three-dimensional reconstruction images obtained by micro-CT were observed to assess Ti particle-mediated bone resorption. As presented in **Figure 1A**, bone destruction and severe osteolysis were clearly discernable with wear particle intervention (vehicle group). In the puerarin treatment groups, however, the severity of osteolysis and bone resorption was significantly alleviated by puerarin treatment. Furthermore, the degree of bone destruction in the high-puerarin-dose treatment group was much better than that in the low-dose group. The white arrows indicate bone osteolysis sites (**Figure 1B**). As depicted in **Figures 1C and D**, quantitative analysis of the relevant parameters further verified that puerarin dramatically promoted BMD and BV/TV in mouse calvariae as puerarin concentration increased. Consistent with BMD and BV/TV, the total porosity and number of pores were clearly inhibited in the puerarin-treated groups compared with the positive control group (**Figures 1E, F**).

**Figure 2:**
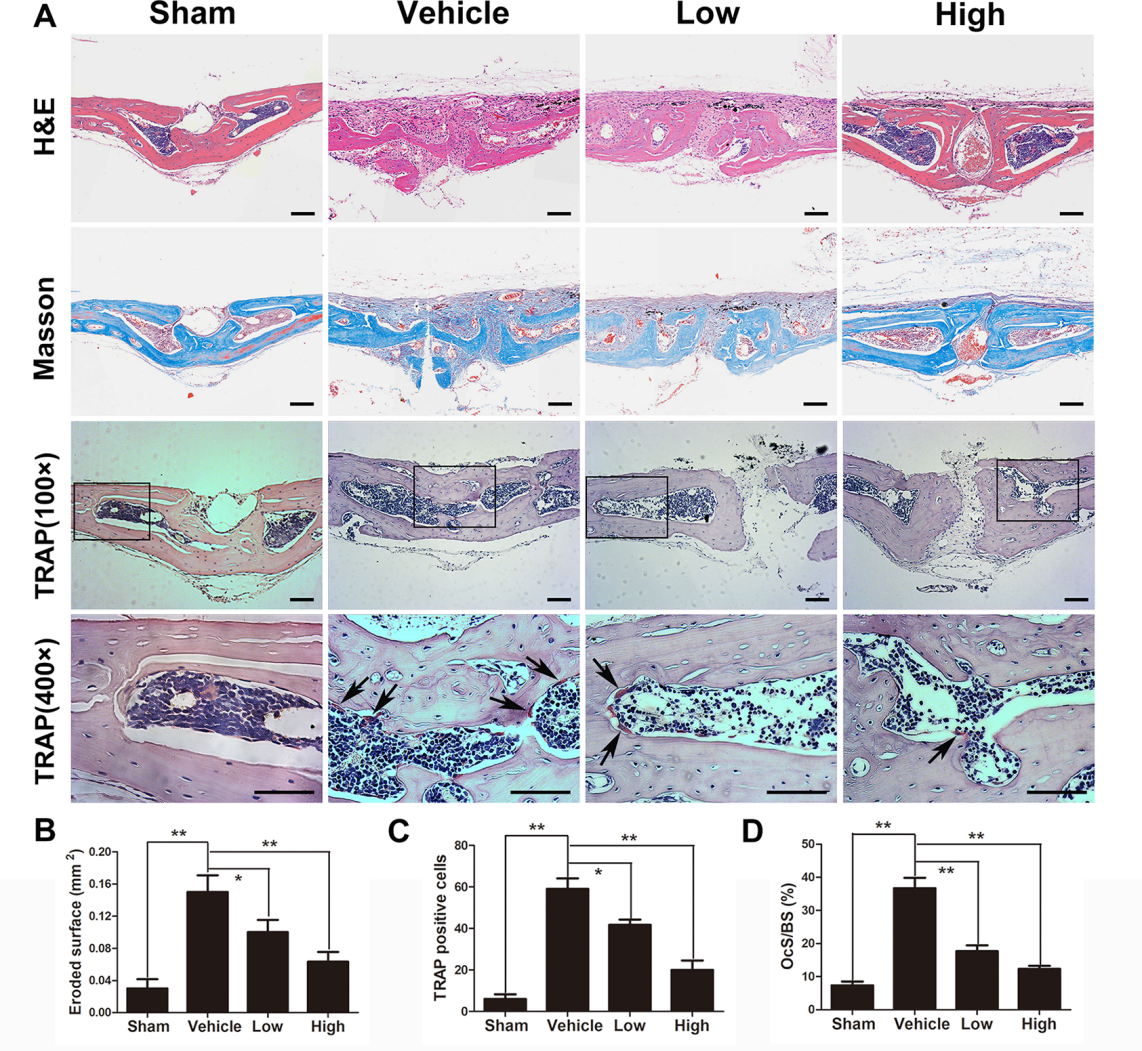
Puerarin prevented Ti particle-induced osteolysis *in vivo*. **(A)** Histological images of H&E-stained, Masson’s trichrome-stained and TRAP-stained (low and high magnification) calvarium sections from each group. TRAP-positive cells are indicated by black arrows. **(B)** Eroded surface area, **(C)** the number of TRAP-positive cells, and **(D)** ratio of osteoclast surface to bone surface (OcS/BS) of each group were measured using Image Pro Plus software 6.0. Data are presented as mean ± SD (n= 4/group); *P < 0.05 and **P < 0.01 compared with the vehicle group. Data are representative of at least three independent experiments. Scale bar = 100 µm.

Histological and immunochemical assessments were further applied to evaluate the protective effects of puerarin on Ti particle-stimulated osteolysis. H&E and Masson’s trichrome staining revealed that obvious bone loss and inflammatory cell infiltration were observed in the wear particles group, whereas when puerarin was injected, these observations were clearly attenuated in the low-dose and high-dose groups (**Figure 2A**). Histomorphometric analysis demonstrated that marked bone erosion occurred with Ti particle treatment. As expected, the puerarin treatment groups displayed significant reductions in surface erosion (**Figure 2B**). TRAP staining further indicated that multinucleated TRAP-positive cells were successfully induced by Ti particles, and the number of TRAP-positive cells was significant decreased in the high-dose puerarin treatment group. In addition, the number of TRAP-positive cells and the OCs/BS were significantly reduced by puerarin intervention, especially the high-dose group (**Figures 2C, D**). The black arrows indicate TRAP-positive cells.

**Figure 3:**
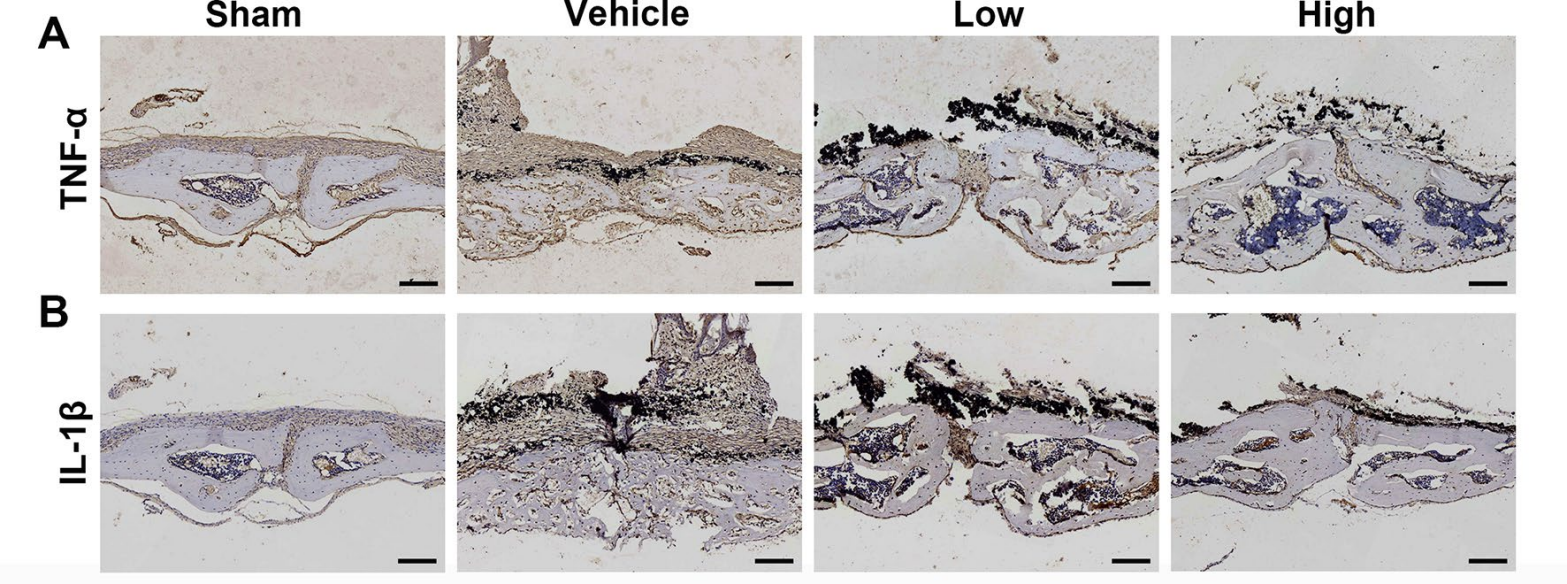
Puerarin reduced inflammatory cytokine expression in Ti particle-induced mouse calvarial osteolysis. **(A**, **B)** Immunohistochemical staining for TNF-α and IL-1**ß** in mouse calvarial of each group. Scale bar = 100 µm.

Immunochemical staining was used to confirm the production of inflammatory cytokines in the wear debris-induced osteolysis model. After treatment with Ti particles, the expression of TNF-α and IL-1β was significantly increased in the vehicle group, whereas proinflammatory cytokines were clearly inhibited in the puerarin-treated groups compared with the positive control group (**Figure 3**). Collectively, these results indicated that puerarin treatment inhibited osteoclast formation during wear debris-mediated bone destruction in a dose-dependent manner *in vivo*.

### Puerarin Alleviated RANKL-Stimulated Osteoclast Formation *in Vitro*

**Figure 4:**
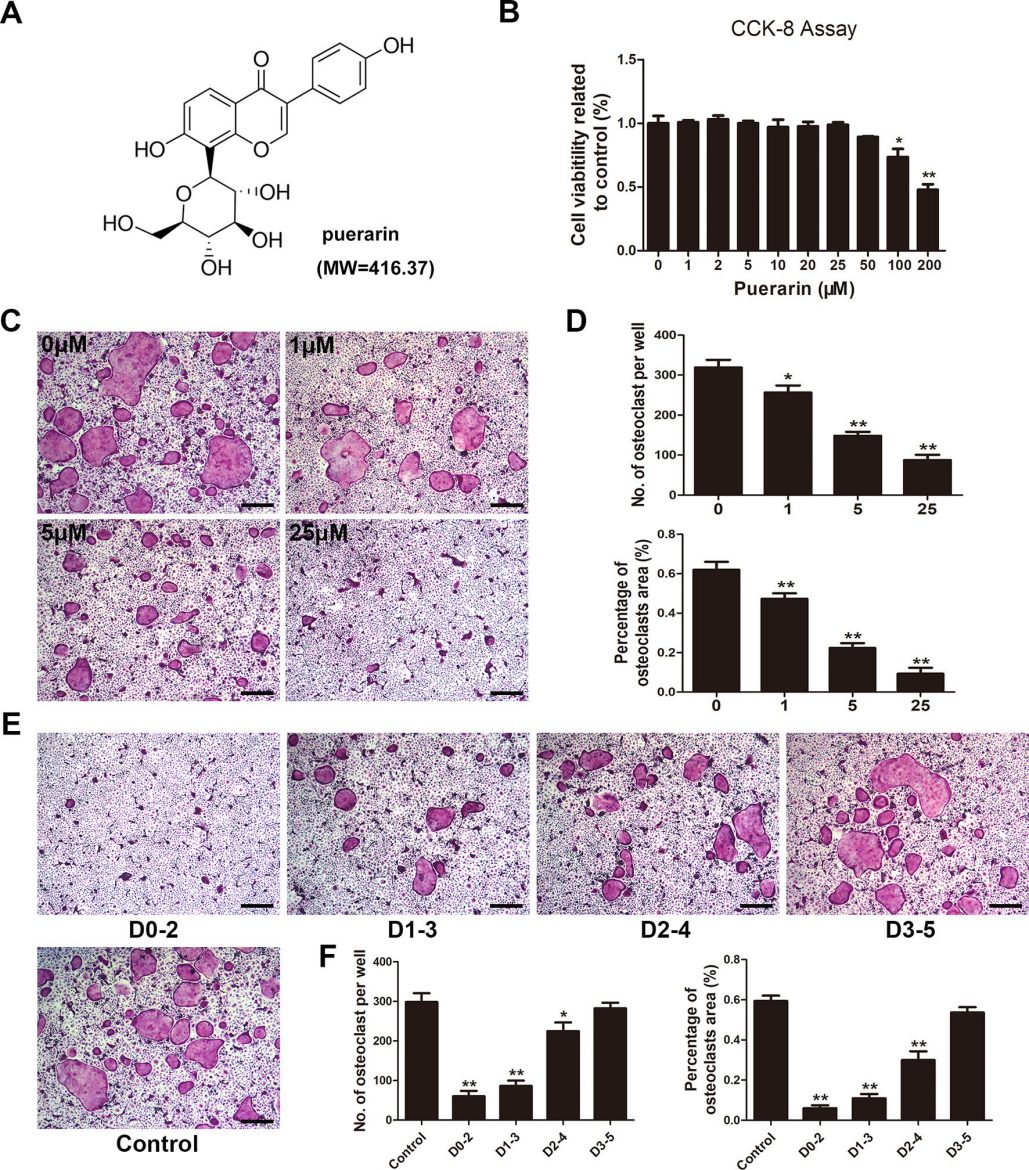
Puerarin inhibited RANKL-induced osteoclast formation *in vitro*. **(A)** Chemical structure of puerarin (MW=416.37). **(B)** Cell viability was evaluated using a Cell Counting Kit (CCK)-8 assay, and the result was normalized to the control group. **(C)** Bone marrow-derived macrophages (BMMs) were cultured and induced in α-MEM medium with the addition of M-CSF (30 ng/ml), RANKL (100 ng/ml) and 0, 1, 5, or 25 µM puerarin, and then subjected to TRAP staining at day 7. **(D)** The number and percentage of TRAP-positive cells under each treatment were quantified. **(E)** BMMs were incubated in media containing 30 ng/ml M-CSF and 100 ng/ml RANKL with 25 µM puerarin from day 0 to 2, day 1 to 3, day 2 to 4 or day 3 to 5, respectively. All BMMs were incubated for 7 days. TRAP staining was performed to analyze the number and percentage of TRAP-positive cells. **(F)** The number and percentage of TRAP-positive cells at each time-point were quantified. Data are presented as mean ± SD; * P < 0.05 and ** P < 0.01 compared with the control group; Data are representative of at least three independent experiments. Scale bar = 100µm.

To evaluate the effect of puerarin on RANKL-mediated osteoclast differentiation, cells were cultured with two osteoclast-stimulated cytokines and various puerarin concentrations. BMMs cytotoxicity and proliferation were evaluated by a CCK-8 assay. No cytotoxicity occurred with puerarin (0–50 μM) in BMMs, indicating that the inhibitory effects of puerarin on bone resorption and osteoclastic function were not induced by cytotoxicity (**Figures 4A, B**). After culturing for 7 days in the presence of puerarin (0, 1, 5, and 25 μM), TRAP staining was conducted to evaluate osteoclast differentiation and showed that typical TRAP-positive cells emerged without the intervention of puerarin (0 μM). However, exposure to puerarin (1, 5, and 25 μM) mitigated the generation of TRAP-positive cells with concentration increased (**Figures 4C, D**). To further confirm whether the early differentiation stage was affected by puerarin, we performed osteoclast differentiation assays with puerarin for a specific period. The TRAP staining results demonstrated that puerarin (25 μM) significantly inhibited osteoclast formation when the BMMs were added on days 0–2 or 1–3. However, this effect was diminished when the cells were treated with puerarin on days 2–4 or 3–5, indicating that puerarin likely inhibited osteoclast formation during the early differentiation stage (**Figures 4E, F**).

### Puerarin Inhibited Osteoclast Function *Via* Suppressing the Generation of F-Actin Rings and Bone Destruction Area

**Figure 5:**
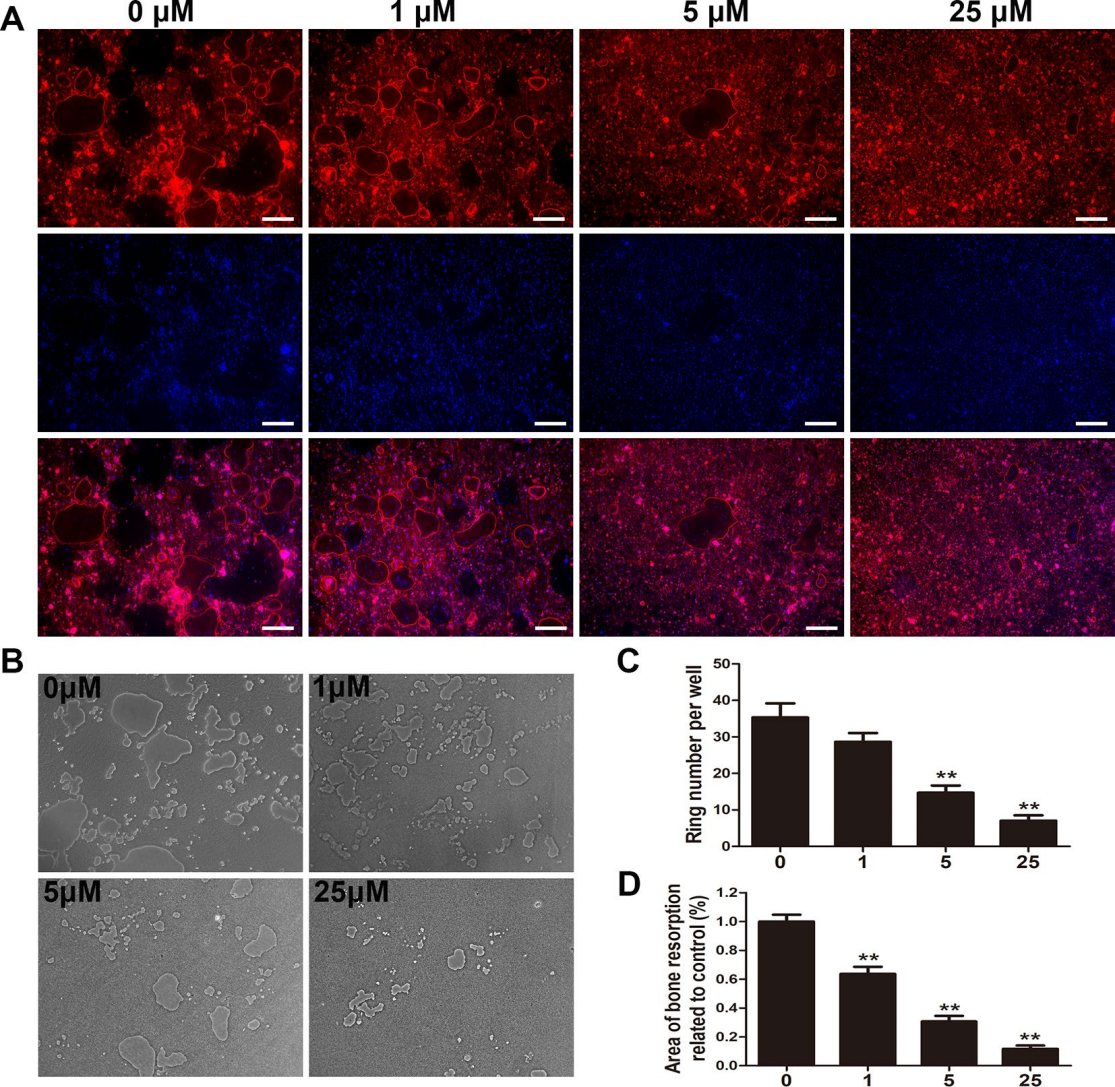
Puerarin inhibited osteoclast fusion and impaired osteoclastic bone resorption *in vitro*. **(A**, **C)** BMMs were induced and cultured in α-MEM medium with the addition of M-CSF (30 ng/mL), RANKL (100 ng/mL) and 0, 1, 5, or 25 µM puerarin for 7 days; then, the cells were stained with phalloidin and DAPI. The number and size of F-actin positive cells were observed using a fluorescence microscope. The number of F-actin rings were quantified by ImageJ software. **(B**, **D)** BMMs were seeded on the Osteo Assay Plate (OAP, Corning, USA) and cultured in osteoclast induction medium for 4 days. When typical osteoclasts were observed on day 4, the osteoclast induction medium with 0, 1, 5, or 25 µM puerarin was replaced and cultured for an additional 3 days. Representative images of bone resorption areas were observed and taken. The area eroded by osteoclasts was quantified by ImageJ software. Data are presented as mean ± SD; **P < 0.01 compared with the control group; Data are representative of at least three independent experiments. Scale bar = 200 µm.

Given that the generation of F-actin rings is critical for osteoclastic function (Wilson et al., 2009), fluorescent staining was applied to verify the potential impact of puerarin on F-actin rings. After staining with rhodamine phalloidin and DAPI, numerous well-organized podosome belts and the formation of typical mature osteoclasts were detected without puerarin intervention, but the addition of puerarin significantly attenuated the size and number of F-actin rings as concentration increased (**Figures 5A, C**).

We further assessed the effect of puerarin on the suppression of bone destruction and resorption using an OAP. Considering the inhibitory effect of puerarin on osteoclastic formation, we surmised that puerarin could also alleviate osteoclastic bone resorption. We observed that the area of bone resorption was significantly mitigated as puerarin concentration increased (**Figures 5B, D**). Therefore, the results indicated that administration of puerarin reduced osteoclastic bone resorption and destruction.

### Puerarin Inhibited Osteoclast-Related Gene Expression *in Vitro*

**Figure 6:**
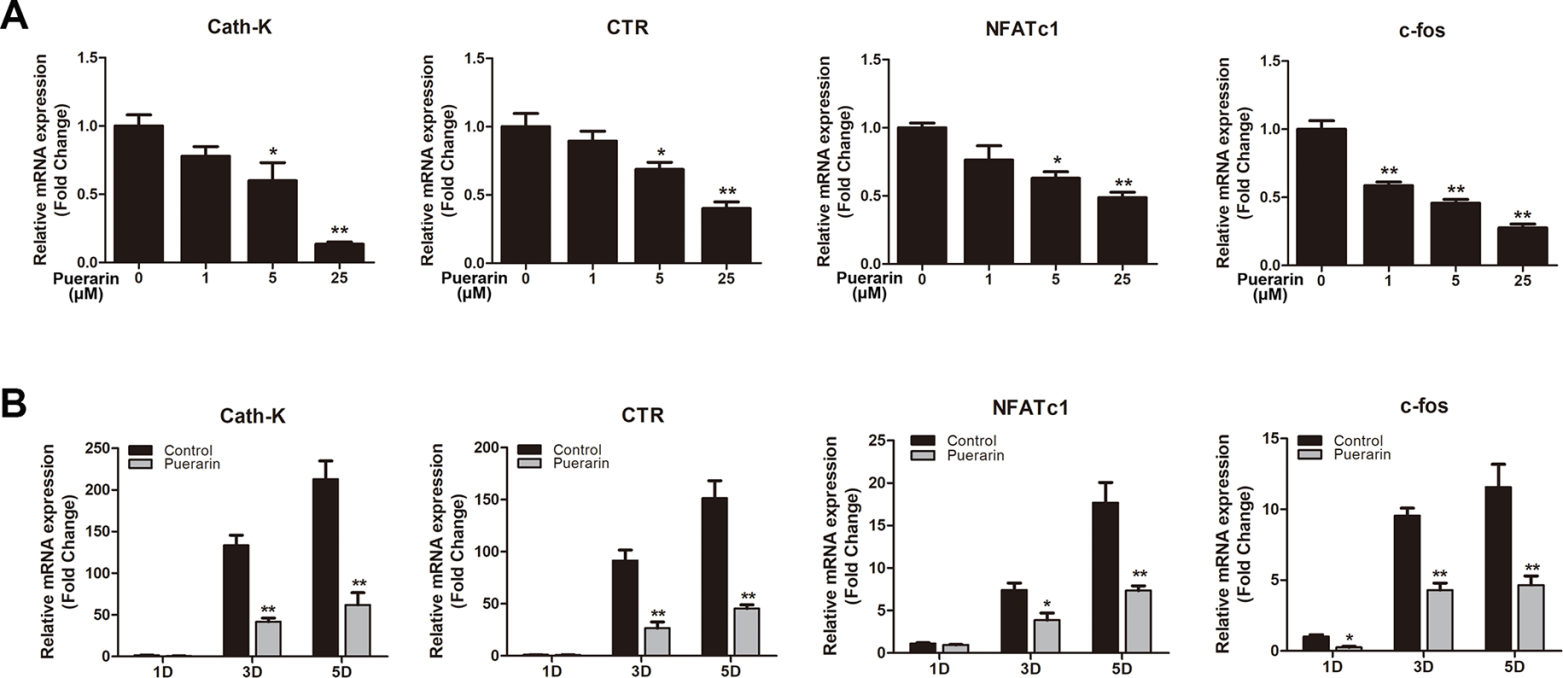
Puerarin downregulated the expression of RANKL-induced osteoclast-related genes including cathepsin K (Cath-K), calcitonin receptor (CTR), c-fos, and nuclear factor of activated T cell cytoplasmic 1 (NFATc1) *in vitro*. **(A)** BMMs were induced in osteoclast induction medium with various puerarin concentrations (0, 1, 5, or 25 µM) for 5 days. **(B)** BMMs were cultured in osteoclast induction medium with or without 25 µM puerarin for 1, 3, or 5 days. The expression of osteoclast-related genes was quantified by RT-PCR. Data are presented as mean ± SD; *P < 0.05 and **P < 0.01 compared with the control group. Data are representative of at least three independent experiments.

Osteoclastic marker gene levels were increased during osteoclast differentiation of BMMs (Boyle et al., 2003). We applied RT-PCR to assess RANKL-stimulated mRNA expression of osteoclastic marker genes in both dose- and time-dependent manners (**Figures 6A, B**). The results demonstrated that RANKL significantly stimulated osteoclast-related gene expression. However, puerarin-treated groups decreased osteoclast-related gene levels as puerarin concentration increased. Furthermore, puerarin (25 μM) also inhibited expression of these gene expression at different stages of osteoclast differentiation. These results further demonstrated that puerarin can suppress osteoclast function and reduce osteoclastic marker gene expression *in vitro*.

### Puerarin Exerted Protective Effects *via* Suppression of the ERK Pathway and the Upstream Regulators MEK1/2

**Figure 7:**
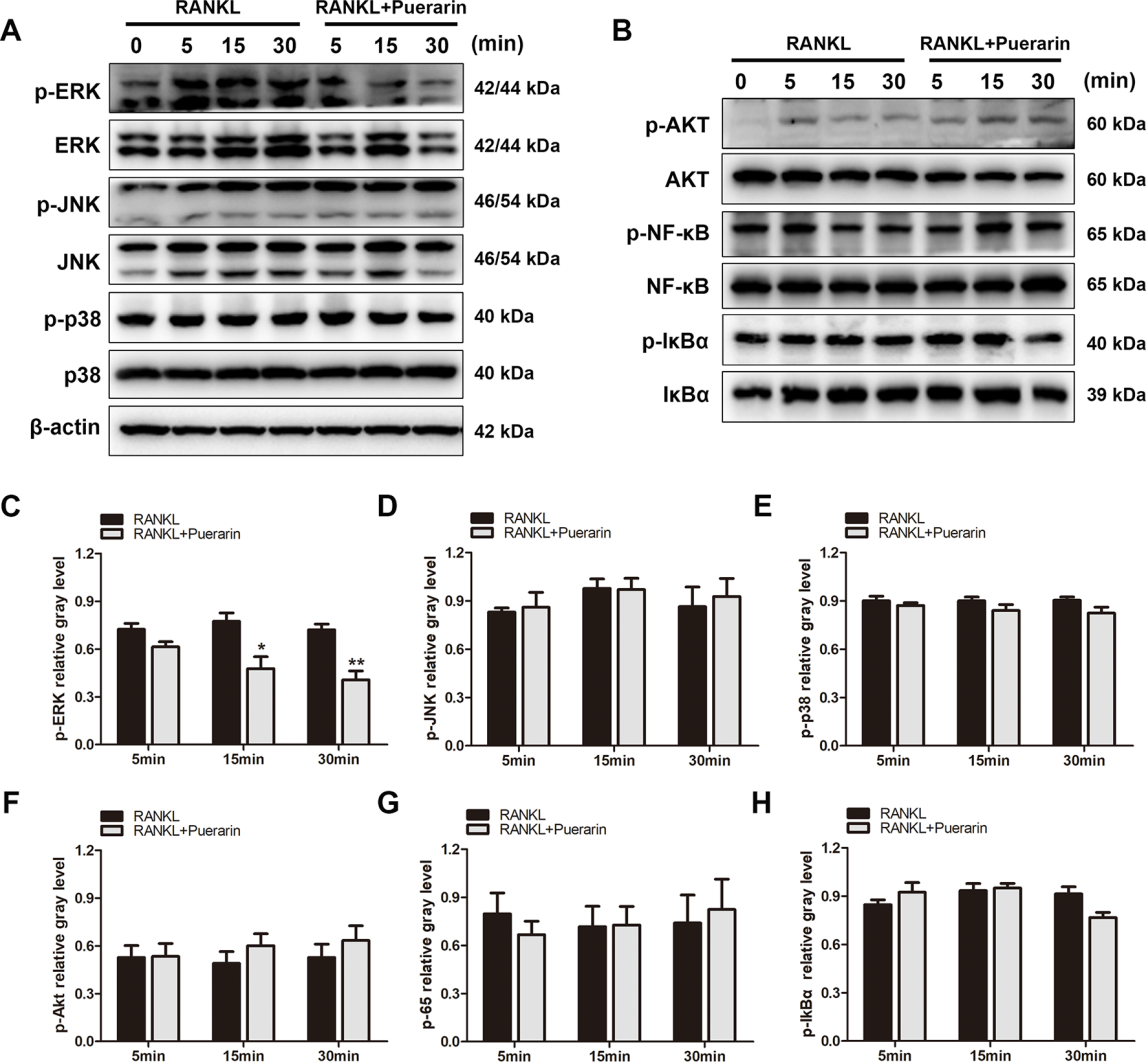
Puerarin suppressed the RANKL-stimulated activation of ERK signaling but did not affect NF-κB or Akt signaling. **(A**, **B)** RAW264.7 cells were pretreated with or without puerarin for 4 h, and then with 100 ng/ml RANKL for indicated time periods (0, 5, 15 or 30 min). Then, the cells were collected and lysed for western blot analysis. **(C**–**H)** The relative gray levels corresponding to p-ERK, p-JNK, p-p38, p-Akt, p-NF-κB and p-IkBα were quantified and normalized to ß-actin using ImageJ software. Data are presented as mean ± SD; *P < 0.05 and **P < 0.01 compared with the control group. Data are representative of at least three independent experiments.

**Figure 8:**
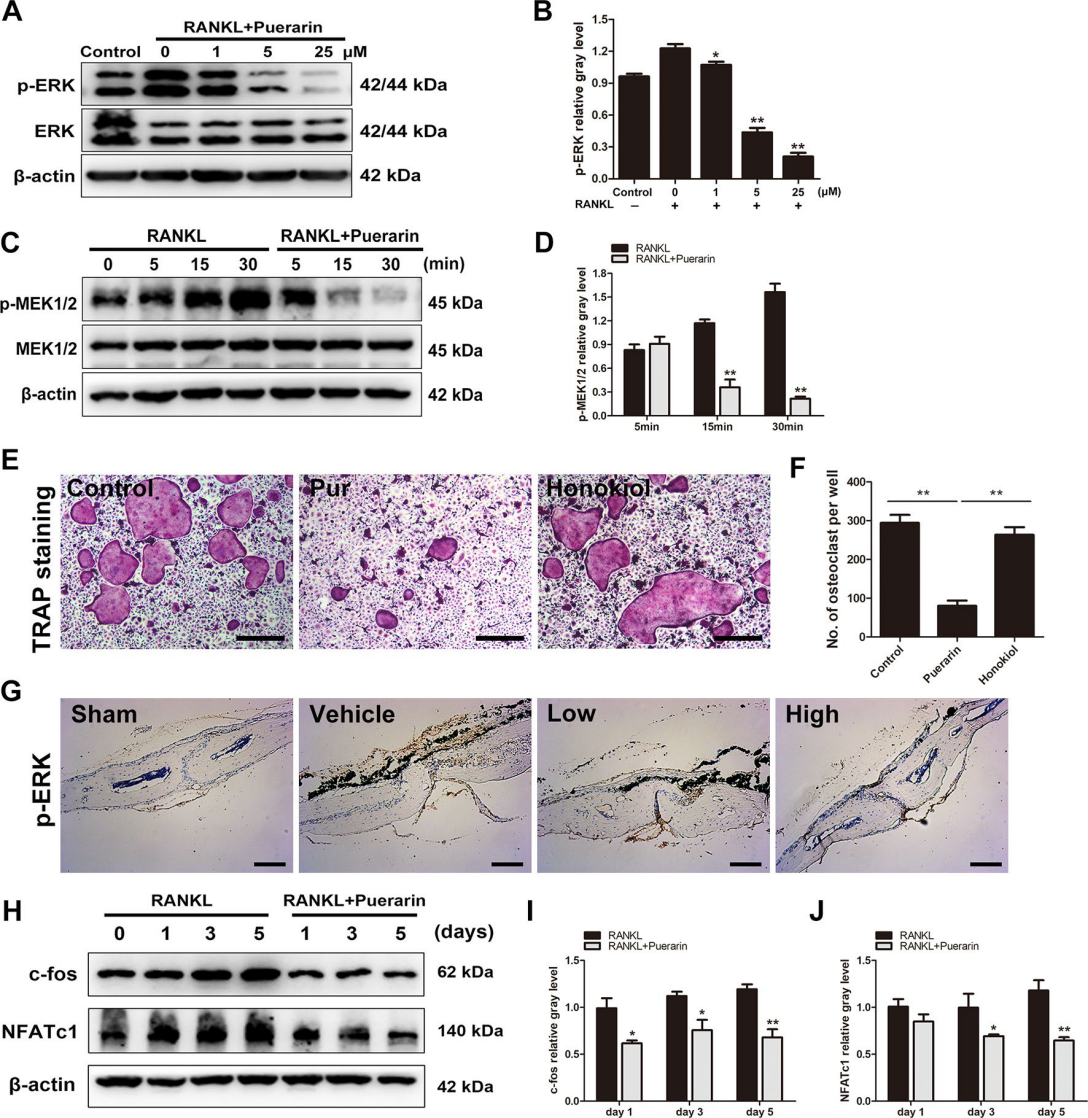
Puerarin attenuated RANKL-mediated osteoclast formation and function via suppressing the activation of MEK1/2, ERK, c-fos and NFATc1 pathways. **(A**, **B)** After pretreatment with various puerarin concentrations (0, 1, 5, or 25 µM) for 4 h. The cells were stimulated with 100 ng/ml RANKL for 15 min. Then, cell lysates were subjected to western blotting against ERK1/2 and p-ERK1/2 antibodies. The relative gray levels corresponding to p-ERK was quantified and normalized to ß-actin using ImageJ software. **(C**, **D)** After pretreatment with or without puerarin (25 µM) for 4 h, the cells were stimulated with 100 ng/ml RANKL for indicated time periods (0, 5, 15 or 30 min). Cell lysates were subjected to western blotting against MEK1/2 and p-MEK1/2 antibodies. The relative gray levels corresponding to p-MEK1/2 was quantified and normalized to ß-actin using ImageJ software. **(E**, **F)** BMMs were pretreated with or without ERK1/2 agonist Honokiol (20 µM) for 12 h, and then with puerarin (25 µM), M-CSF (30 ng/ml) and RANKL (100 ng/ml) for 7 days. Cells were fixed and stained for TRAP. The number of TRAP-positive cells under each treatment were quantified. **(G)** Immunohistochemical staining for ERK in mouse calvarial of each group. **(H)** After pretreatment with or without puerarin (25 µM) for 4 h, the cells were cultured in induction medium for 0, 1, 3 or 5 days. Cells were then collected and lysed for western blot analysis. **(I**, **J)** The relative gray levels of corresponding to c-fos and NFATc1 were quantified and normalized to ß-actin using ImageJ software. Data are presented as mean ± SD; *P < 0.05 and **P < 0.01 compared with the control group. Data are representative of at least three independent experiments. Scale bar = 100 µm.

To define the potential mechanisms through which puerarin exerts an inhibitory effect on osteoclastic precursor cells differentiation, several relevant pathways were evaluated, including the PI3k/Akt, NF-κB, and MAPK pathways (Asagiri and Takayanagi, 2007; Yuan et al., 2015; Wu et al., 2018b). After pretreatment with or without puerarin (25 μM), the BMMs were cultured with RANKL for a specific period to identify the activation of the signaling molecules involved. Several studies have demonstrated that the subfamilies of ERK, JNK, and p38 in MAPK pathways play a crucial role in osteoclast differentiation from osteoclast precursor cells (Tai et al., 2014). Interestingly, the results indicated that puerarin reduced ERK phosphorylation at 15 min and 30 min, but this was not observed with the JNK or p38 pathways (**Figures 7A, C–E**). Furthermore, this inhibitory effect was also enhanced in a dose-dependent manner (**Figures 8A, B**). However, puerarin showed no inhibitory effects on RANKL-induced p65 activation or IkBα degradation, suggesting that the intervention of puerarin exerted no effect on the NF-κB pathways (**Figures 7B, G, H**). Similarly, puerarin had no significant influence on the activation of the PI3k/Akt pathways (**Figures 7B, F**).

We further investigated whether puerarin directly affected the ERK pathway, and the upstream regulator was also explored. Studies have demonstrated that ERK1/2 was activated by the closely related MEK1/2, which is an upstream regulator of the ERK pathway(Hu et al., 2017; Wu et al., 2018b). Results demonstrated that puerarin treatment (25 μM) alleviated RANKL-stimulated phosphorylation of MEK1/2 (**Figures 8C, D**). Furthermore, the specific ERK agonist Honokiol was used in the culture system. We observed that the inhibitory effect of puerarin was reversed by ERK agonist Honokiol (**Figures 8E, F**). Immunochemical staining of ERK *in vivo* further confirmed that the expression of p-ERK was significantly increased in the vehicle group, whereas p-ERK expression were clearly inhibited in the puerarin-treated groups compared with the positive control group (**Figure 8G**). Given that puerarin attenuates ERK phosphorylation and the consequently influences the expression of c-fos and NFATc1 (Takayanagi et al., 2002; Yasui et al., 2011), we further investigated whether puerarin suppressed the upregulated expression of downstream transcription factors to induce osteoclastogenesis. Cells were seeded and cultured with or without puerarin (25 μM) for 1, 3, and 5 days. We observed that c-fos and NFATc1 were markedly alleviated by puerarin intervention (**Figures 8H–J**). Therefore, these results demonstrated that puerarin mitigated osteoclast function and formation by suppressing the activation of RANKL-mediated MEK1/2, ERK, c-fos and NFATc1.

## Discussion

Artificial joint replacement is considered the most successful operation for end-stage joint diseases. However, the major postoperative complication for artificial joints is aseptic loosening, which is mainly induced by wear debris from artificial implants (Holt et al., 2007; Rao et al., 2012). Nevertheless, the secretion of various proinflammatory cytokines is essential to osteoclast formation, and excessive osteoclast formation and function are considered as the main causes of osteolysis (Shao et al., 2015). Previous studies have demonstrated that wear debris-mediated inflammatory reactions and the consequent activation of osteoclast function commonly occur in osteolytic diseases (Harris, 2001; Gallo et al., 2013). Therefore, there is an urgent need for a new strategy to attenuate wear debris-mediated inflammation in osteolysis by suppressing osteoclast function and differentiation. In this work, we demonstrated that puerarin alleviates wear debris-stimulated bone resorption in a murine calvarial osteolysis model, and this inhibitory effect was regulated *via* suppression of the RANKL-mediated ERK pathway during the process of osteoclast formation and maturation.

Studies have verified the primary effect exerted by osteoclasts in inflammatory osteolysis and subsequent aseptic loosening (Hotokezaka et al., 2007; Ping et al., 2017a). This work initially confirmed that puerarin could suppress wear particle-stimulated bone destruction in a calvarial osteolysis model. Our study provides evidence that puerarin significantly reduced the number of osteoclasts and surface erosion compared to those in the Ti particles group. Furthermore, the results of histological and immunohistochemical staining also demonstrated that puerarin exerted a protective effect on inflammatory reactions and osteolysis. Subsequently, in the *in vitro* cell model, puerarin reduced the bone resorption area as concentration increased, and osteoclast formation and function were significantly suppressed during the early differentiation stage. In addition, we further verified puerarin treatment downregulated the mRNA levels of osteoclastic marker genes, such as CTR, Cath-K, NFATc1, and c-fos, in dose- and time-dependent manners. Collectively, these findings demonstrated that puerarin exhibits a protective effect on wear debris-mediated osteolysis by suppressing osteoclast differentiation and osteoclastic function.

Next, we investigated the potential mechanisms of the inhibitory effect exerted by puerarin on osteoclast differentiation. RANKL-mediated activation of the PI3k/Akt and NF-κB pathways is crucial for osteoclast function. Previous research demonstrated that puerarin suppresses lipopolysaccharide-induced osteoclast differentiation by Akt phosphorylation (Zhang et al., 2016). However, the PI3k/Akt and NF-κB pathways did not appear to be activated by puerarin pretreatment. This discrepancy in results may be due to different effects by the stimuli RANKL and lipopolysaccharide. Further, the MAPK pathway also played a crucial role in osteoclast differentiation and function. ERK, JNK, and p38 are members of the MAPK pathway family, and RANKL can rapidly stimulate the phosphorylation of these subfamilies (Tai et al., 2014). Studies have suggested that the MAPK families are involved in osteoclast differentiation, growth, and function (Miyazaki et al., 2000). In the MAPK signaling pathway, ERK exerts a significant effect on the function and survival of osteoclastic precursor cells. JNK or p38 also participate in osteoclast formation, whereas the p38 pathway has no effect on bone resorption (Pearson et al., 2001; Jiang et al., 2018). In our study, puerarin was found to inhibit the phosphorylation of ERK, but not other subfamilies, indicating that puerarin may serve as a specific regulator specific of ERK pathway. Furthermore, the phosphorylation of MEK1/2, upstream regulators of the ERK pathway, was also affected by puerarin. Along with suppressed ERK pathway activation, the downstream osteoclastic transcription factors c-fos and NFATc1 were also markedly reduced following puerarin treatment. A previous study verified that these transcription factors played crucial roles in regulating the early differentiation stage of osteoclast precursor cells. Furthermore, as a part of the AP-1 transcription factor complex, c-fos was activated by ERK pathway phosphorylation (Teitelbaum, 2004). NFATc1 is a transcription site in osteoclast-related signaling pathways, controlled by c-fos and regulates osteoclastic marker gene levels (Asagiri et al., 2005; Lu et al., 2014). This evidence indicates that NFATc1 possesses an indispensable function in osteoclast differentiation. Collectively, the underlying mechanism explores in this study demonstrates that puerarin attenuates osteoclast function and osteoclastic marker gene levels by inhibiting the MEK1/2, ERK, and NFATc1 pathways.

Interestingly, studies have shown that resveratrol and other polyphenols also alleviate wear particle-stimulated osteolysis as well as some neurodegenerative diseases resulting from inflammatory response and oxidative stress (Chillemi et al., 2015; Luo et al., 2016; Miquel et al., 2018). The pharmacological modulation of inflammation and oxidation systems might serve as a viable approach for preventing and treating inflammatory diseases (Calabrese et al., 2018; Peng et al., 2019). Further, we also found that puerarin, as previously observed with resveratrol, plays a pharmacological role within the hermetic non-linear dose-response model, which is characterized by low-concentrations activation and high-concentration inhibition (Calabrese et al., 2016; Wang et al., 2018). The results showed that low puerarin concentrations (0–50 μM) exhibited no cytotoxic effects on cells, indicating that the inhibitory effect of puerarin on osteoclast formation and function was not induced by cytotoxicity. The hermetic-dose response may be one reason that puerarin inhibited the wear debris-mediated osteolysis within the effective concentration range. Given these potential properties of polyphenols, puerarin may also be applied in the treatment of other diseases, such as neurodegenerative disorders. Therefore, these findings further indicated that puerarin, as well as other polyphenols, exert a protective effect in inflammatory diseases, which may contribute to its anti-inflammatory and antioxidant properties and the hermetic-dose response.

Furthermore, some limitations remain in our work. Firstly, the primary wear particles that induce osteolysis are ultra-high molecular weight polyethylene particles (UHMWPE) rather than Ti debris. However, both of these particles have been shown to equally mimic authentic wear debris and cause inflammatory bone destruction. Therefore, other osteolytic initiation agents should also be investigated to verify the beneficial effect of puerarin. Secondly, various cells (osteoblasts, fibroblasts, and other mesenchymal cells) participate in the development of wear particle-mediated osteolysis and puerarin may exhibit different effects in different cells and environments. Thirdly, studies have indicated that the production of ROS, NO, and Ca^2+^ oscillation may also influence the activation of osteoclastic differentiation and function. Moreover, puerarin exerted more pharmacological effects on osteoclast formation during early stages than were observed in later ones. The results indicated that puerarin may possess an important role in preventing osteolysis at early stages, and it will be necessary to perform a systematic investigation of this effect produced by puerarin in wear particle-induced osteolysis in our future research.

In conclusion, this study demonstrated that puerarin significantly alleviated wear particle-mediated bone destruction in a mouse calvarial model and mitigated osteoclastic precursor cell differentiation and function at a cellular level, presumably *via* suppression of the MEK/ERK pathways and its downstream factors. We therefore propose that puerarin might serve as an effective agent in the prevention of osteoclastic diseases such as wear particles-stimulated osteolysis.

## Data Availability Statement

All datasets generated for this study are included in the manuscript and the **Supplementary Files**.

## Author Contributions

XZ and YQ designed this study. KZ, XY and TC helped revise the manuscript. All authors reviewed the manuscript and approved the final manuscript.

## Funding

This study was supported by the National Natural Science Foundation of China (81772309, 81673998), Shanghai Pujiang Talent Program (18PJD035), Scientific Research Project of Shanghai Municipal Health Commission (201540151).

## Conflict of Interest

The authors declare that the research was conducted in the absence of any commercial or financial relationships that could be construed as a potential conflict of interest.
