# Supplementary material for: Puerarin Exerts Protective Effects on Wear Particle-Induced Inflammatory Osteolysis
Source: Front Pharmacol. 2019 Oct 1;10:1113. doi: 10.3389/fphar.2019.01113 (PMC6779862; doi:10.3389/fphar.2019.01113)
Supplement: Supplementary file 1 [file DataSheet_1.docx]

**Puerarin Exerts Protective Effects on Wear Particle-Induced Inflammatory Osteolysis**

***Chao Yang^1^, Juehong Li^1^, Kechao Zhu, Xiangwei Yuan, Tao Cheng,*** ***Yebin Qian* and Xianlong Zhang****

*Department of Orthopedics, Shanghai Jiao Tong University Affiliated Sixth People’s Hospital, Shanghai 200233, China*

**Corresponding Authors:*

*Xianlong Zhang: dr_*[*zhangxianlong@163.com*](mailto:zhangxianlong@163.com)

*Yebin Qian:* [*yebinqian@aliyun.com*](mailto:yebinqian@aliyun.com)

The characteristics of Ti particles were evaluated by transmission electron microscope (TEM). The morphology of the Ti particles was observed by TEM. The characteristics of Ti particles were presented in **Figure S1**. Most Ti particles were of irregular morphology and flock together, which were findings similar to the wear particles as previously described.


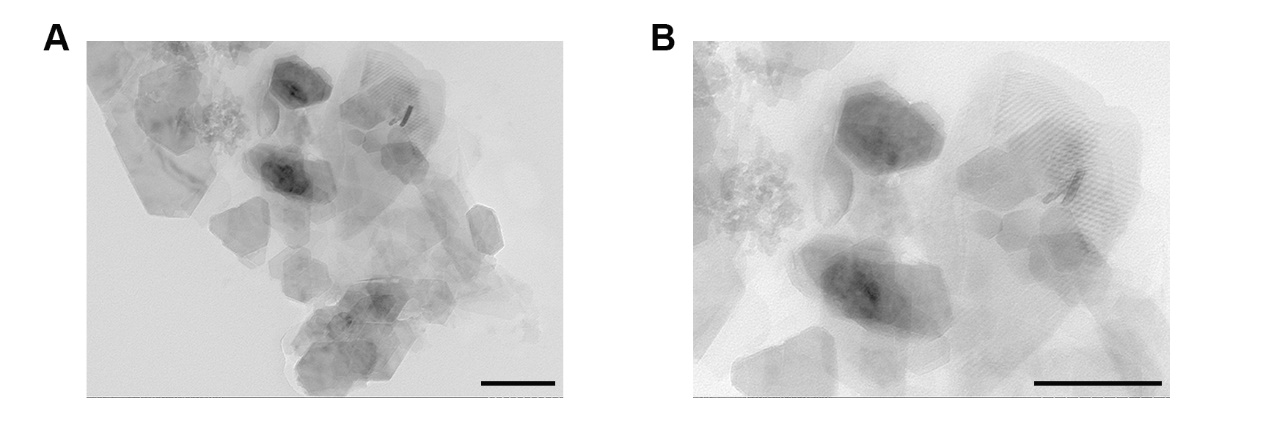


**Figure S1.** Characterizations of titanium (Ti) particles were observed by transmission electron microscope (TEM). **(A, B)** Representative TEM images of Ti particles. Scale bar = 200 nm

The results indicated that puerarin reduced the phosphorylation of ERK pathway. Another two protein bands of p-ERK were presented in **Figure S2.**


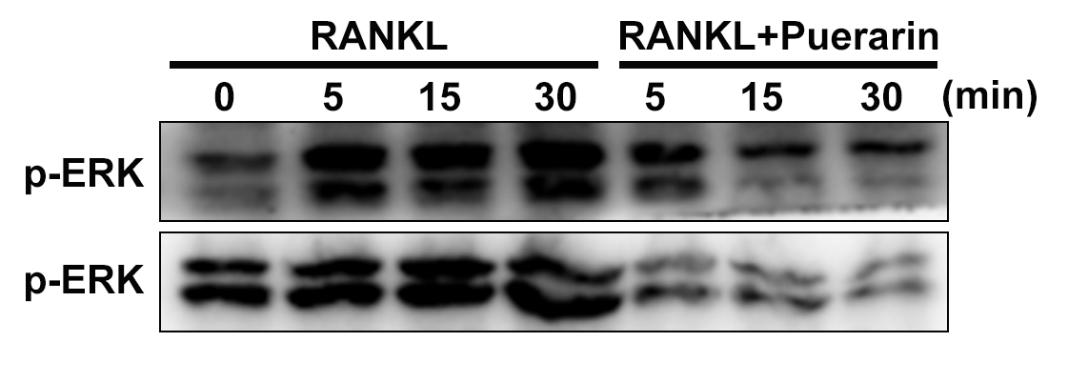


**Figure S2.** Puerarin suppressed the RANKL-stimulated activation of ERK signaling pathway.
